# Thyroid metastasis from esophageal adenocarcinoma: a case report and literature review

**DOI:** 10.1186/s40792-019-0695-5

**Published:** 2019-08-30

**Authors:** Shinsei Yumoto, Yoshifumi Baba, Daichi Nomoto, Kazutaka Oozono, Kojiro Eto, Yukiharu Hiyoshi, Yohei Nagai, Masaaki Iwatsuki, Shiro Iwagami, Yuji Miyamoto, Naoya Yoshida, Yoshiki Mikami, Hideo Baba

**Affiliations:** 1 Department of Gastroenterological Surgery, Graduate School of Medical Sciences, Kumamoto University, 1-1-1 Honjo, Chuo-ku, Kumamoto, 860-8556 Japan; 2 Department of Diagnostic Pathology, Kumamoto University Hospital, 1-1-1 Honjo, Chuo-ku, Kumamoto, 860-8556 Japan

**Keywords:** Thyroid metastasis, Esophageal cancer, Barrett’s esophageal adenocarcinoma

## Abstract

**Background:**

The incidence of metastatic spread of gastrointestinal malignancies to the thyroid gland is relatively low and most of these malignancies originate from the colorectum. Thyroid metastasis originating from the esophagus is poorly documented.

**Case presentation:**

A 79-year-old man presented with hoarseness of voice and swallowing difficulty. Eighteen months earlier, he had undergone preoperative chemotherapy (S-1 and oxaliplatin [SOX] therapy) and subtotal esophagectomy with regional lymph nodes dissection and retrosternal narrow gastric tube reconstruction for advanced Barrett’s esophageal adenocarcinoma. In the ultrasonographic examination, there was a hypoechoic, indistinct border and heterogeneous nodule in the left lobe of the thyroid gland. Pathological examination of an ultrasound-guided fine-needle aspiration showed adenocarcinoma, supporting a diagnosis of esophageal adenocarcinoma metastases in the thyroid.

**Conclusion:**

This is a first case of a patient with thyroid metastasis from Barrett’s esophageal adenocarcinoma after subtotal esophagectomy.

## Background

The incidence of metastatic spread of gastrointestinal malignancies to the thyroid gland is relatively low, and most of these malignancies originate from the colorectum [1]. Thyroid metastasis originating from the esophagus is poorly documented. Herein, we report a first case of a patient with thyroid metastasis from Barrett’s esophageal adenocarcinoma after subtotal esophagectomy.

## Case presentation

**Fig. 1:**
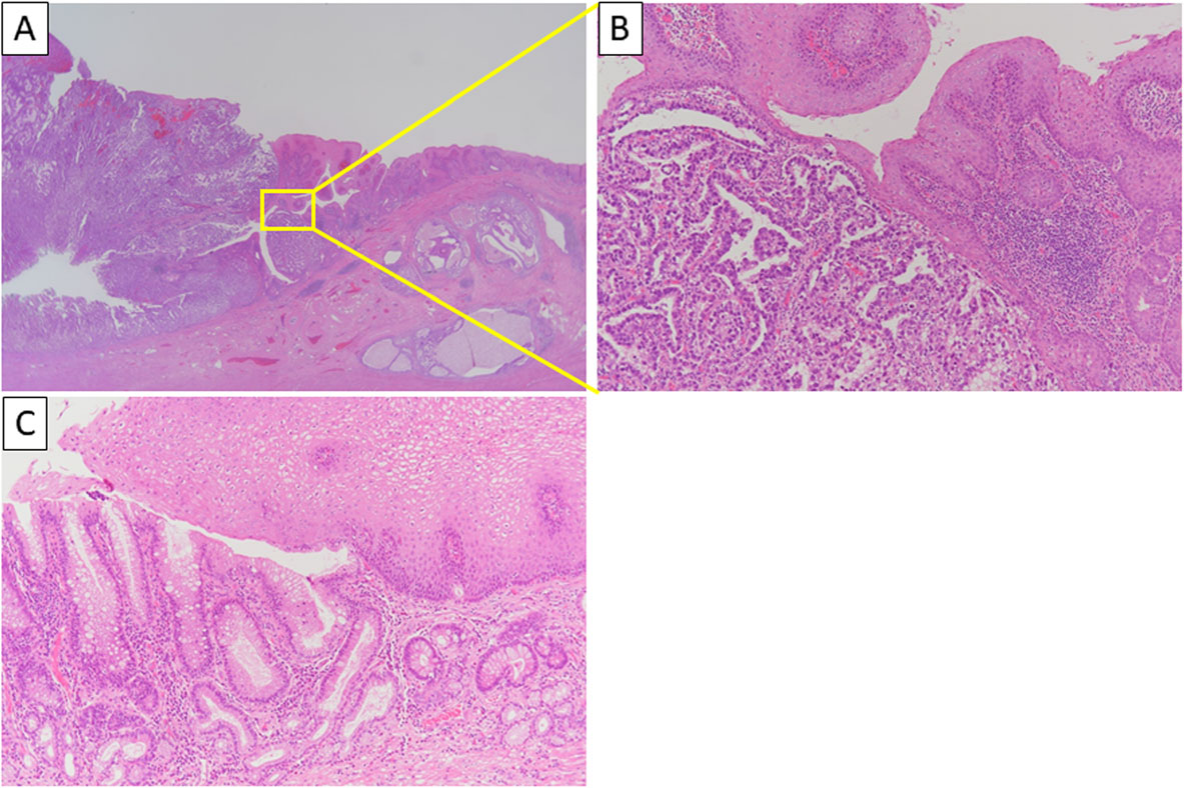
Hematoxylin-eosin staining of the resected specimen. **a** Adenocarcinoma is present at the esophagogastric junction (× 20 magnification). **b** A moderately differentiated adenocarcinoma is present at the esophagogastric junction (× 200 magnification). **c** Distal esophageal squamous epithelium is replaced by specialized columnar epithelium with goblet cells (× 200 magnification)

A 79-year-old man presented with hoarseness of voice and swallowing difficulty. Eighteen months earlier, he had undergone preoperative chemotherapy (S-1 and oxaliplatin [SOX] therapy) and subtotal esophagectomy with regional lymph node dissection and retrosternal narrow gastric tube reconstruction for advanced esophageal cancer. The histological examination of the resected specimen revealed Barrett’s esophageal adenocarcinoma pStage III (8th UICC TNM classification) (Fig. 1).

**Fig. 2:**
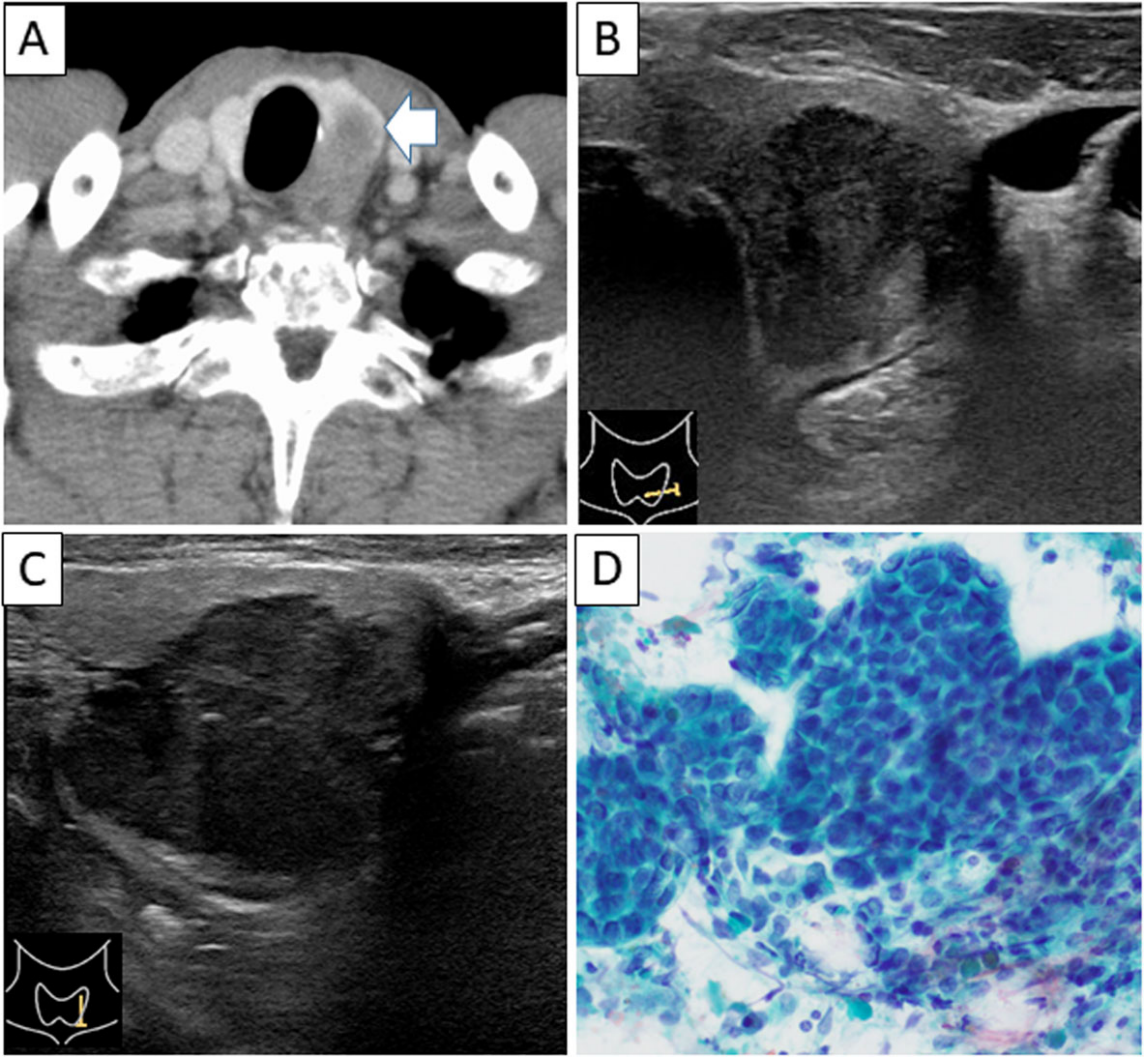
Imaging and pathological findings. **a** Contrast-enhanced computed tomography (CT) revealed a low-density mass in left thyroid gland. **b**, **c** Ultrasonographic (US) examination showed a hypoechoic, indistinct border and heterogeneous nodule measuring 16.9 mm × 19.7 mm × 23.9 mm in the left lobe of the thyroid gland. **d** Pathological examination of an ultrasound-guided fine-needle aspiration showed adenocarcinoma

Upper gastrointestinal endoscopy showed no abnormal finding including anastomosis site (cervical esophagus and gastric tube). Contrast-enhanced computed tomography (CT) revealed a low-density mass in his left thyroid gland (Fig. 2a). In the ultrasonographic examination, there was a hypoechoic, indistinct border and heterogeneous nodule measuring 16.9 mm × 19.7 mm × 23.9 mm in the left lobe of the thyroid gland (Fig. 2b, c). Pathological examination of an ultrasound-guided fine-needle aspiration showed adenocarcinoma, supporting a diagnosis of esophageal adenocarcinoma metastases in the thyroid (Fig. 2d). The patient commenced chemotherapy with pembrolizumab, combined chemotherapy of docetaxel with cisplatin (CDDP), and 5-fluorouracil (5-FU) in a clinical trial. This treatment effectively shrank the tumor after seven courses.

## Discussion

The incidence of intrathyroidal metastases in autopsy series varies from 1.25 to 24.0% in cancer patients [2, 3]. The metastatic spread of gastrointestinal malignancies to the thyroid gland is relatively rare; the majority originate from the colorectum [1]. Thyroid metastasis from the esophagus has only been reported in eight cases in the English-language literature [1, 4–11]. Importantly, this is the first case of thyroid metastasis from Barrett’s esophageal carcinoma.

Table 1 summarizes the clinical features of the eight previously published cases plus our report of thyroid metastasis from esophageal cancer. The age of the patients at presentation was variable, ranging from 32 to 79 years with an average age of 62.1 years. Four out of the eight previously reported cases were treated with thyroidectomy, and the management in the other two cases was not reported [12]. In the postoperative histopathological specimen, six patients showed squamous cell carcinomas and two were adenocarcinomas. Thyroid metastasis of Barrett’s adenocarcinoma has never been reported previously in any literature.

**Table 1:** The literature review of cases with thyroid metastasis from esophageal cancer

Source	Age	Sex	Treatment for thyroid	Pathology result	Outcomes
Present case	79	M	Chemotherapy	Adenocarcinoma	4 months (alive)
Shuangshoti [4]	58	M	TT + ipsilateral CL	SCC	11 months (dead)
Yamada et al. [5]	74	F	ST + bilateral CL	SCC	Over 4 years (alive)
Basu et al. [6]	55	F	No data	SCC	No data
Cumbo-Nacheli et al. [7]	32	M	No data	Adenocarcinoma	No data
Moulick et al. [8]	66	M	Chemoradiation	SCC	No data
Chen et al. [9]	61	M	Palliative bilateral NT + tracheostomy	SCC	11 months (dead)
Cheng et al. [10]	70	M	Right lobectomy + partial left lobectomy	SCC	3 months (dead)
Reese et al. [11]	64	M	None	Adenocarcinoma	No data

*TT* total thyroidectomy, *NT* near-total thyroidectomy, *ST* subtotal thyroidectomy, *CL* cervical lymphadenectomy, *SCC* squamous cell carcinoma

Generally, despite being second to the adrenal glands as the most vascular perfused organ in the body [13], the thyroid is rarely considered to be the sole site of metastases in the clinical practice and is usually asymptomatic [14, 15]. Cichon et al. reported that metastasis to the thyroid only accounts for 2 to 3% of all thyroid carcinomas identified in the clinical practice [16]. The most common primary sites are the kidney, breast, and lung [17–20]. Direct extension of adjacent primaries, a hematogenous pathway, and lymphatic route for metastatic spread to the thyroid have been estimated [6, 21]. Czech et al. suggested that the vertebral vein plexus may play an important role in the process of metastases from other organs to the thyroid [19]. Unfortunately, according to a review of the related literature, no case of careful imaging and pathologic evaluation of the most likely route of metastasis in the thyroid has been reported. In our case, the tumor may be considered as a lymphogenous metastasis because there were cervical lymph node metastases in the postoperative histopathological specimen and this lymph node was anatomically close to the thyroid. Of course, we acknowledge that further examination such as autopsy may be useful to clarify the mechanism.

There is no clear consensus on therapeutic strategy for metastatic thyroid cancers from esophageal cancer. Thus, the management is determined on a case-by-case basis [1, 9, 22]. Overall, most patients with metastasis to the thyroid had poor outcomes, with reported 9-month survival after the original diagnosis [23] (Table 1). However, one case was reported where the patient was without evidence of recurrence 4 years after thyroidectomy [5]. In the present case, the patient commenced chemotherapy with pembrolizumab, CDDP, and 5-FU in a clinical trial and experienced a favorable therapeutic effect. If the tumor is still reduced with no new metastatic lesion for some time, we might consider performing surgical resection (i.e., thyroidectomy).

## Conclusions

This case highlights the need for awareness of the possibility of potential metastatic deposits in unexpected sites. A new thyroid mass with a history of cancer, however remote the previous primary cancer was, should be evaluated for the possibility of metastasis. Metastasis should also be strongly considered whenever the histology is unusual for a primary thyroid lesion. Although the prognosis of metastasis in the thyroid is commonly poor, patients may have improved quality of life and longer survival time after early accurate diagnosis and proper treatment.

## Data Availability

All data generated or analyzed during this study are included in this published article.

## Abbreviations

SOX: S-1 and oxaliplatin
UICC: Union for International Cancer Control
CT: Computed tomography
CDDP: Cisplatin
5-FU: 5-Fluorouracil
TT: Total thyroidectomy
NT: Near-total thyroidectomy
ST: Subtotal thyroidectomy
CL: Cervical lymphadenectomy
SCC: Squamous cell carcinoma
